# Cardiorespiratory Fitness in Occupational Groups—Trends over 20 Years and Future Forecasts

**DOI:** 10.3390/ijerph18168437

**Published:** 2021-08-10

**Authors:** Daniel Väisänen, Lena. V. Kallings, Gunnar Andersson, Peter Wallin, Erik Hemmingsson, Elin Ekblom-Bak

**Affiliations:** 1 Department of Physical Activity and Health, The Swedish School of Sport and Health Sciences, 10316 Stockholm, Sweden; lena.kallings@gih.se (L.V.K.); erik.hemmingsson@gih.se (E.H.); elin.ekblombak@gih.se (E.E.-B.); 2 Health Profile Institute, 18211 Danderyd, Sweden; gunnar.anderssont@hpihealth.se (G.A.); peter.wallin@hpihealth.se (P.W.)

**Keywords:** white-collar, blue-collar, VO_2_max, fitness, occupational groups, cardiorespiratory fitness, trends, forecast, prediction

## Abstract

Background: Reports have indicated a negative trend in cardiorespiratory fitness (CRF) in the general population. However, trends in relation to different occupational groups are missing. Therefore, the aim of our study was to examine the trends in CRF during the last 20 years, and to provide a prognosis of future trends in CRF, in different occupational groups of Swedish workers. Methods: Data from 516,122 health profile assessments performed between 2001 to 2020 were included. CRF was assessed as maximal oxygen consumption and was estimated from a submaximal cycling test. Analyses include CRF as a weighted average, standardized proportions with low CRF (<32 mL/min/kg), adjusted annual change in CRF, and forecasting of future trends in CRF. Results: There was a decrease in CRF over the study period, with the largest decrease in both absolute and relative CRF seen for individuals working in administrative and customer service (−10.1% and −9.4%) and mechanical manufacturing (−6.5% and −7.8%) occupations. The greatest annual decrease was seen in transport occupations (−1.62 mL/min/kg, 95% CI −0.190 to −0.134). Men and younger individuals had in generally a more pronounced decrease in CRF. The proportion with a low CRF increased, with the greatest increase noted for blue-collar and low-skilled occupations (range: +19% to +27% relative change). The forecast analyses predicted a continuing downward trend of CRF. Conclusion: CRF has declined in most occupational groups in Sweden over the last two decades, with a more pronounced decline in blue-collar and low-skilled occupational groups.

## 1. Introduction

Cardiorespiratory fitness (CRF) has long been reported as a strong indicator of health, non-communicable diseases, and life expectancy [1]. Two of the main determinants of the CRF level are current moderate-to-vigorous physical activity (MVPA) and heredity [2,3,4,5]. Of these two, only the MVPA level is modifiable, and it is the main alternative to maintain or increase CRF.

Low socioeconomic status, often assessed using education or income, has been linked to lower levels of both MVPA and CRF [6,7]. Along with the higher prevalence of daily smoking, poor diet, and obesity, the risk of common diseases such as cardiovascular diseases and type-2 diabetes is higher in those with low compared with high socioeconomic status [8,9].

We recently compared lifestyle-related risk indicators between different occupational groups, where blue-collar and low-skilled occupations had a higher prevalence of obesity, daily smoking, and poor diet [10]. However, interestingly, the proportion of participants with low CRF differed between occupations, despite having a similar level of education. This means that the assessment of CRF in relation to occupation, compared with other measures of socioeconomics, can provide additional important aspects in relation to health and disease risk, especially as a certain level of CRF is required for the job tasks of several occupations [11]. On the contrary, in more sedentary occupations (office workers), low CRF has been associated with a lower cognitive performance and higher sickness absenteeism [12,13].

While the importance of CRF for health and disease risk is well established, less is known regarding the trends in CRF over the recent decades in the general population, as well as in sub-groups of the population. Some reports indicate a negative trend in CRF in the general population [14,15], with a more pronounced decrease in men, younger age groups, and in those with low education. However, the trends in relation to different occupational groups are missing. Such information would be highly relevant in order to identify and target interventions towards occupations that need it the most.

The aim of this study was to examine the secular trends in CRF over the last 20 years in different occupational groups of the Swedish working population, and to forecast possible future trends.

## 2. Methods

**Figure A1:**
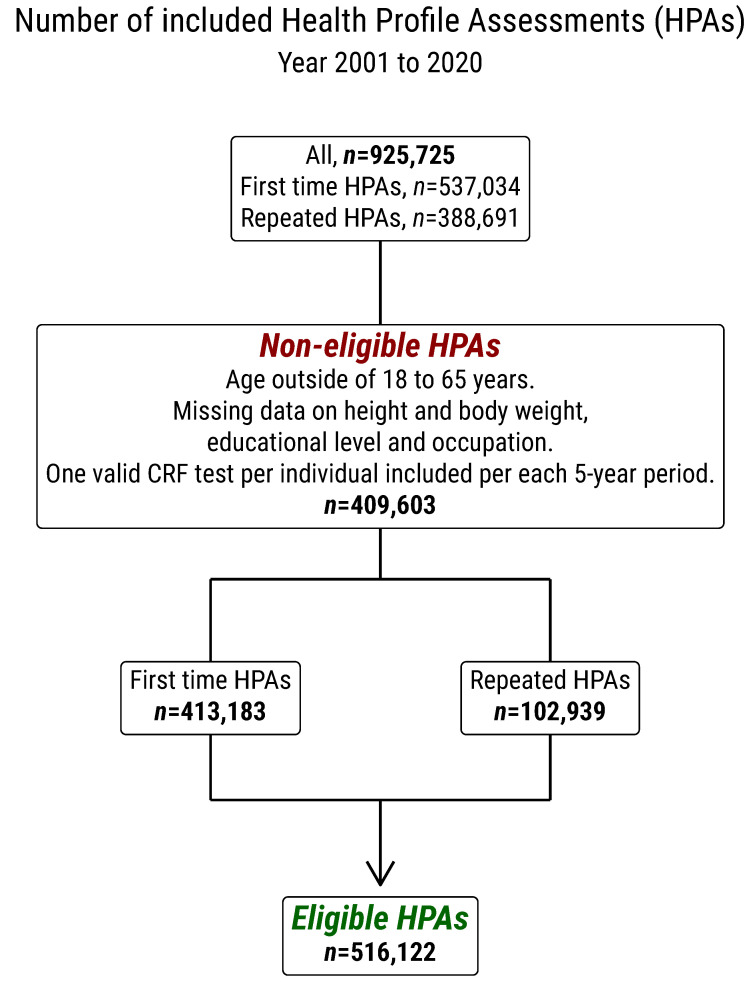
Flow chart.

Data were taken from Health Profile Institute’s database (assessed on 1 December 2020) that contained data from Health Profile Assessments (HPA) that were carried out in health services all around Sweden since the 1970s. HPA is an interdisciplinary method consisting of a questionnaire regarding lifestyle and health experiences, measurements of anthropometrics and blood pressure, a submaximal cycle test for estimation of maximal oxygen consumption (VO_2_max), and dialogue with a Health Profile Coach. Participation is offered to all employees in a company or organization connected to occupational or health services and is free of charge. The HPA method has been developed and standardized throughout the years by the HPI Health Profile Institute, which is also responsible for the database. HPAs have been available in the database from 1980. However, for power reasons, we included data from January 2001 to December 2020 in the present analyses, aggregated into five-year periods (2001–2005, 2006–2010, 2011–2015, and 2016–2020). During this period, a total of 925,725 HPAs were carried out, of which 537,034 were first time HPAs and 388,691 were repeated HPAs in the same individuals (see Figure A1 for flow chart). In participants with repeated HPAs, one valid CRF test per individual was included in each five-year period. Inclusion criteria for the present analyses were age between 18 and 65 years, data on height and body weight, educational level, and occupation. The final filtered sample included 516,122 tests, of which 413,183 were first time tests and 102,939 were repeated tests. Participants provided informed consent prior to data collection. The study was approved by the ethics board at the Stockholm Ethics Review Board (Dnr 2015/1864-31/2 and 2016/9-32) and adhered to the Declaration of Helsinki.

### 2.1. Assessment of Estimated VO_2_max

The estimated VO_2_max was obtained from the Åstrand submaximal cycle ergometer test [16]. The participants were requested to refrain from vigorous activity the day before the test, consuming a heavy meal 3 h before, smoking/snuff use 1 h before the test, and avoiding stressing during the test. The test was conducted on a calibrated cycle ergometer at an individually adapted submaximal work rate for 6 min. VO_2_max was estimated from the achieved steady state pulse using a sex specific nomogram, with corresponding age-correction factors, and was expressed as the absolute (L/min) and relative (mL/min/kg) VO_2_max [16]. A low CRF was defined as <32 mL/min/kg, as it has been reported as an inflection point of increased risk of mortality [17]. The Åstrand test has shown a low variation in the mean difference between the estimated and directly measured VO_2_max (mean difference −0.07 L/min 95% CI −0.21 to 0.06) [18].

### 2.2. Occupational Groups

Out of the 516,122 individual occupational codes, 248,467 were reported by the participants at the HPA, and they were coded according to the Swedish Standard Classification of Occupation (SSYK) [19]. The remaining SSYK codes were derived from the national register data (Statistics Sweden: www.scb.se). These were based on SSYK reported by the employer and were matched for the year of the HPA performed. Each occupation was derived from a four-digit SSYK-code; the first digit defined the major group, the second digit defined the sub-major group, the third digit defined the minor group, and the fourth digit defined the unit group. Here, we present data on the major and sub-major levels. The major level is presented as one digit, while the sub-major is presented as a digit and a decimal digit. The occupational groups with corresponding SSYK codes included in the present analyses were as follows (1) managers; (2.1) science and engineering; (2.2) health care; (2.3) education; (2.4) other professionals; (3) associate professionals; (4) administrative and customer service; (5) service, care, and shop sales; (7) building and manufacturing; (8.1) mechanical manufacturing; (8.2) transport; and (9) elementary occupations. Furthermore, we used aggregated occupational categories for some analyses, defined as white-collar high-skilled (major groups 1–3), white-collar low-skilled (major groups 4 and 5), blue-collar high-skilled (major group 7), and blue-collar low-skilled (major groups 8 and 9). Major group 6 of agriculture and forestry was excluded due to a low number of participants (*n* = 3624). A more detailed description of the occupational groups can be acquired from Vaisanen et al. [10].

### 2.3. Other Measurements

The highest educational attainment at the time of the HPA was derived from Statistics Sweden (www.scb.se, assessed on 01 December 2020) by linking the participant personal identity number, and was defined as length of education, ≤8 years, =9 years, =10 years, =12 years, =13–15 years, ≥16 years, and ≥17 years/research education, and was further aggregated for different analyses (see the Statistics section). Body mass and height were measured at the HPA in light-weight clothes, to the nearest 0.5 cm and 0.5 kg, with standardized equipment. Age was limited to 18 to 65 years and was, together with sex, derived from the HPI data.

### 2.4. Statistics

Weighted average was used to study the trends in both absolute (L/min) and relative (mL/min/kg) mean CRF in the 12 occupational groups, using the aggregated five-year period variable. Weights were calculated from the frequency of individuals according to sex, age-groups (18–34, 35–49, and 50–65), and education group (≤11 years, 12 years, and ≥12 years) in the Swedish population in 2019. Each value of CRF was multiplied by the assigned weight, which was summed and divided by the number of data points.

To calculate the average difference per year in CRF, three different linear regression models were used for each occupational group—a main effects model and two interaction models (the latter with year*sex and year*age-group). All of the models were controlled for year performed as a continuous variable, age-group, sex, education as a seven-pointed scale (≤8 years, =9 years, =10#x2013;11 years, =12 years, =13–15 years, ≥16 years, and ≥17 years/research education) and weight.

We calculated the potential influence of weight gain by dividing the difference between the decrease in relative and absolute CRF (in %) by the decrease in relative CRF. Contrasts were used to compare sex and age-group differences. All of the *p*-values were corrected with respect to the number of models using Bonferroni correction.

Direct standardization was used to study the trends in low CRF (<32 mL/min/kg) in four aggregated occupational groups (white-collar high skilled, white-collar low-skilled, blue-collar high skilled, and blue-collar low-skilled). The reference weights were calculated from the Swedish population in 2019 according to education (low ≤ 11 years or high ≥ 12 years), sex, and age-group in five-year categories. Confidence intervals were calculated with the normal approximation method.

The forecast was based on data from a weighted average calculated for two-year groups (the first two-year group of 2001–2002 and the last two-year group of 2019–2020). Several different Holt models, arima, naïve drift, and linear trend, as well as combinations of these models were tested for constant variance over time and autocorrelation. The models were tested in cross-validation, tuning the model parameters to get the lowest root mean squared error. The resulting model with the lowest root mean squared error was an ensemble model based on Holts linear exponential smoothening with a trend and a naïve model. The ensemble model was a close approximation of a linear extrapolation, which is reasonable given the approximately linear nature of the data.

All of the analyses and graphics were made using R (version 4.0.5, Austria, Vienna), with the packages Tidyverse [20], Emmeans [21], and Fable [22].

## 3. Results

The characteristics of the participants are shown in Table 1. There were a greater number of participants with HPA in the last 10 years (*n* = 327,213) compared with the first 10 years (*n* = 188,909) of the study period. The proportion of women decreased from 52% to 35% from the first to the last five-year period, while the age-group constitutions were evenly distributed throughout time. The proportion of participants with a high education increased, whereas the participants with a low education decreased. Body weight increased by 4.2 kg (6.1%) in women and 3.6 kg (4.3%) in men between 2001 and 2020. The proportion of blue-collar high-skilled participants increased over time, whereas the proportion of white-collar low-skilled participants decreased.

**Figure 1:**
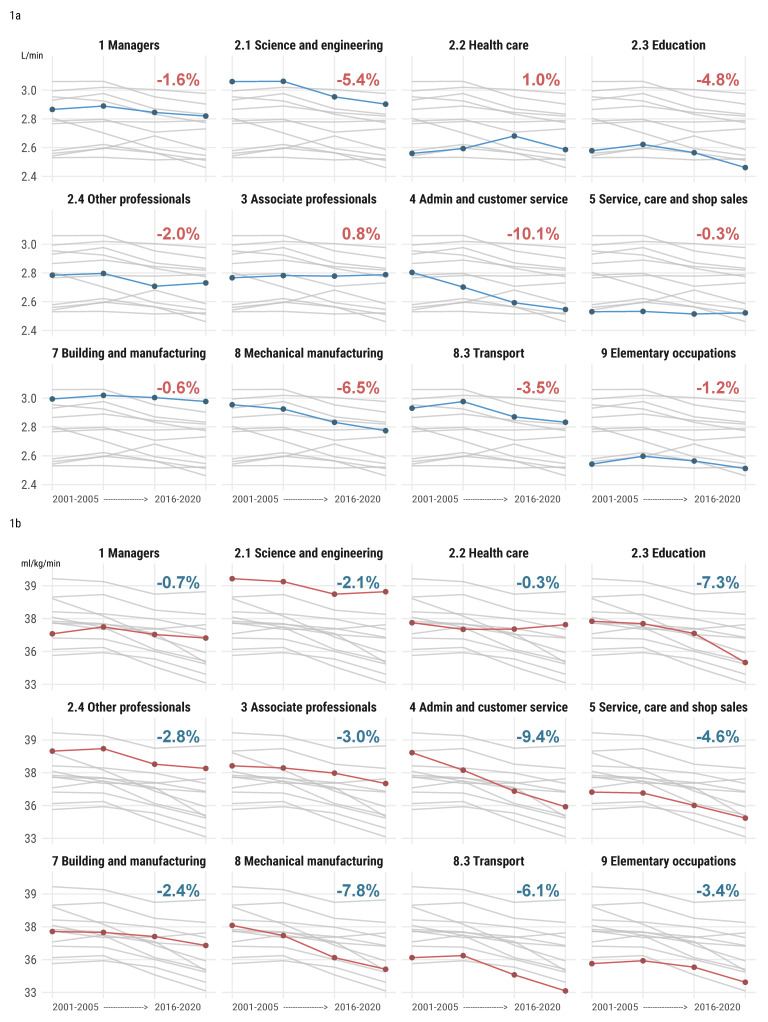
(**a**) Change in absolute CRF (L/min) and (**b**) relative CRF (mL/min/kg) in different occupational groups between 2001 and 2020. Gray lines show all other occupational groups. Percentages shown in the figure are change in CRF between the first and last 5-year period.

The weighted trend values in the absolute and relative CRF are shown in Figure 1a,b, respectively. The largest decreases in both the absolute and relative CRF between the first and last five-year period were seen in administrative and customer service (absolute VO_2_max 2.8 L/min to 2.6 L/min, *p* < 0.001, −10.1% decrease, and relative VO_2_max 38.4 mL/min/kg to 34.9 mL/min/kg, *p* < 0.001, −9.4% decrease). In addition, a large decrease was seen in mechanical manufacturing (absolute VO_2_max 3.0 L/kg to 2.8 L/kg, *p* < 0.001, −6.5% decrease, and relative VO_2_max 37.1 mL/min/kg to 34.4 mL/min/kg, *p* < 0.001, −7.8% decrease). A large decrease in absolute CRF was seen in science and engineering (3.1 L/min to 2.9 L/min, *p* < 0.001, −5.4% decrease). Large decreases in the relative CRF were seen in transport (35.1 mL/min/kg to 33.1 mL/min/kg, *p* < 0.001, −6.1% decrease) and building and manufacturing (36.7 mL/min/kg to 35.9 mL/min/kg, *p* < 0.001, −2.4% decrease). Only marginal changes were present in the absolute CRF for associate professionals (+0.8%, *p* < 0.001) and service, care, and shop sales (−0.3%, *p* < 0.001), and in relative CRF for managers (*p* = 1.000) and health care (*p* < 0.001). For relative CRF, it was for managers (−0.7%, *p* = 0.036) and health care (−0.3%, *p* = 1.000).

The larger decrease in relative compared with absolute CRF in seven of the occupational groups may have been attributed to simultaneous weight gain (as weight influences relative CRF). Approximately one third of the decrease in relative CRF may be explained by weight for education (34% due to weight gain) and other professionals (29%), while similar calculations revealed a larger contribution in some occupational groups (service care and shop sales, 94%, transport, 43%, and elementary occupations, 65%), and less for mechanical manufacturing (17%).

**Figure 2:**
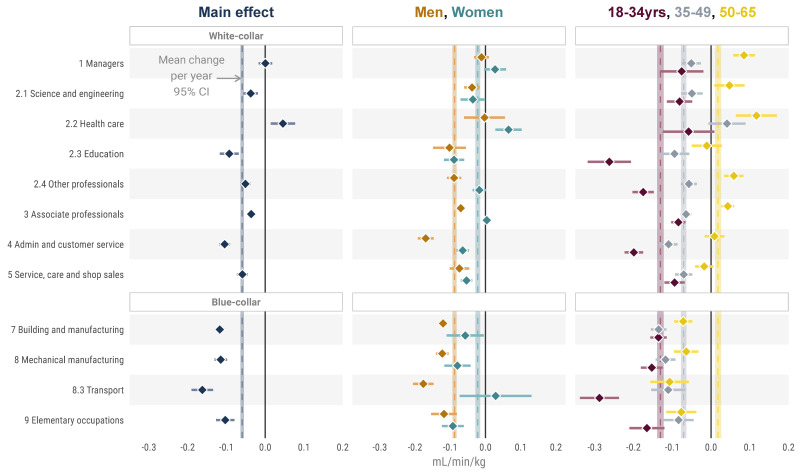
Change in CRF per year in the different occupational groups, both in the total study population and in relation to sex and age-group. Vertical lines are mean annual change in CRF in the total study population (black, thin line) and in relation to each sex and age-group (same color as assigned to each sub-group, respectively).

Regression models presenting change per year, adjusted for age, sex, weight, and education, are shown in Figure 2 and Table A1, Table A2 and Table A3. The main effect model (detailed in Table A1) shows no change in CRF per year in managers (adjusted β = 0.000 (−0.017 to 0.018), *p* = 1) and an increase for health care (adjusted β = 0.046 (0.014 to 0.077), *p* = 0.180) in CRF, while transport (β = −0.162 (−0.190 to −0.134), *p* < 0.001) stood out with the largest negative change per year. While white-collar occupational groups had a large range in change point estimates (β = −0.105 to 0.045) in different occupational groups, blue-collar occupational groups were more homogenous (β = −0.162 to −0.103).

For other professionals, associate professionals, administrative and customer service, and transport, there were significant interactions between men and women (adjusted *p* < 0.001), with a more pronounced decrease per year in men compared with women for all (Table A4).

There was a clear distinction in change in CRF per year when comparing the youngest and the oldest age-groups, where the younger group had a larger decrease in all individual occupational groups, *p* < 0.001 (Table A5). This was specifically pronounced in education, other professional, administrative and customer service, and transport. For managers, science and engineering, health care, other professionals, and associate professionals, the oldest age-group had an increase in CRF.

**Figure 3:**
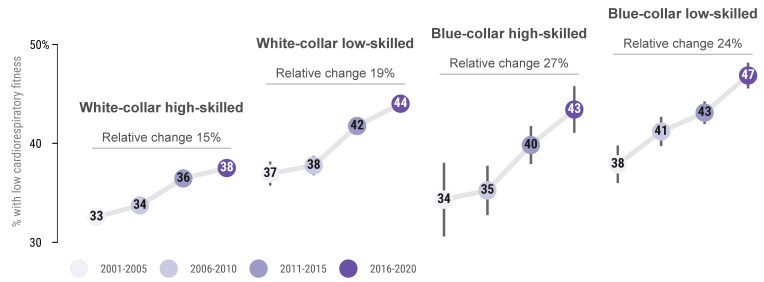
Proportion with, and relative change of proportion with low CRF (<32 mL/min/kg) by aggregated occupational groups over the study period.

Figure 3 shows the change in proportion of low CRF (<32 mL/min/kg) over the study period in relation to the aggregation of occupational groups. All of the aggregated groups had an increase in the proportion with a low CRF. White-collar high-skilled occupations had the lowest proportion of low CRF at all timepoints and the lowest increase over the time period.

**Figure 4:**
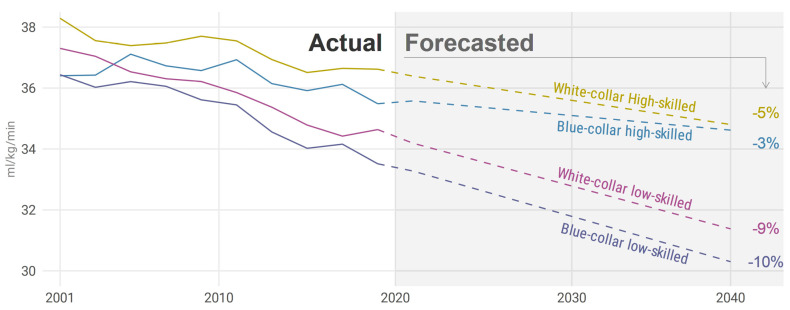
Actual (2001–2020) and forecasted (until 2040) trends in CRF in relation to the aggregated occupational groups. Relative percentages shown in the figure are for the forecasted period.

In the forecast analysis, the trend of decreasing CRF continued in all occupational groups into the foreseeable future (Figure 4). The forecast model predicts a relatively large decrease in blue-collar low-skilled, −10% (from 33.3 mL/min/kg, to 30.3 mL/min/kg), and white-collar low-skilled, −9% (from 34.2 mL/min/kg, to 31.4 mL/min/kg), until 2040. The forecast predicts a smaller decrease in both white-collar high-skilled, −5% (from 36.4 mL/min/kg, to 34.8 mL/min/kg), and blue-collar high-skilled, −3% (from 35.6 mL/min/kg, to 34.6 mL/min/kg).

## 4. Discussion

We noted a large and consistent overall decrease in CRF between 2001 and 2020. However, this decrease was not uniform across occupational groups, largely following a social gradient. Administrative and customer service and mechanical manufacturing occupational groups had the most pronounced decrease in both absolute and relative CRF, with a large decrease in relative CRF also seen for education and transport occupations. Managers had no change and health care had a positive change. The proportion with low CRF increased in all aggregated occupational groups, with a greater (at all time points) increase in the blue-collar and low-skilled occupational groups. Forecast analyses revealed a continued downward trend of CRF, especially in low-skilled occupations (both white-collar and blue-collar).

### 4.1. Earlier Research

There is limited research on CRF levels in different occupational groups beyond comparing white- and blue-collar workers [23,24,25,26,27,28], with even less data on the trends in CRF in different occupational groups. We previously presented a decrease in CRF of −10.8% in the present study population between 1995 and 2017 [14], with a more pronounced decrease in men, young age, and short education. These results are further diversified in the present paper. A systematic review of the change in CRF internationally between 1967 and 2016 showed a mean decrease per decade of 1.6%, with steeper change in recent years, and with a slightly larger decline in men and in younger age-groups aligning at all points with the present study results, where we had a relative mean decrease per decade of 2.1% or 0.73 mL/min/kg [15].

Physical activity of a moderate and vigorous intensity (hereby referred to as exercise) may contribute to maintained and/or increased CRF [1]. According to a Norwegian study, exercise increased in all age-groups (30 and 89 years) from 2001 to 2016, and similar patterns have been seen in other studies [29,30,31]. However, this is self-reported data and can be subjected to biases such as over- or under-estimation of true physical activity levels and a possible changed perception of physical activity over time caused by social desirability [32,33]. In addition, technical development and new transportation options may have reduced the need to be active in everyday life, making exercise the main way to accumulate MVPA. One sensor-based study in a Swedish sample showed a decrease in physical activity between two measurements in 2002 and 2008 [34]. This data, based on a more objective measure, a more valid and reliable data source, are more in line with the present results. Furthermore, in the U.S., U.K, Brazil, and China, downward trends in total physical activity from 1965/2000 until 2005/2009 have been reported, and are projected to decline until 2030, with a large part of the decrease consisting of a decrease in occupational physical activity [35].

### 4.2. Absolute and Relative CRF—The Role of weight Gain

The difference between relative and absolute CRF in percent can be viewed as the impact of weight gain on the change in relative CRF. In our sample, all but managers and science and engineering had a higher decrease in relative compared with absolute CRF—the decrease in relative CRF in these occupational groups may be due to both decreased aerobic capacity and to weight gain. This is highly clinically relevant, as a lower CRF and higher BMI have independently been associated with increased morbidity and mortality [36,37]. While the highest risk of mortality was observed in those who were both obese and unfit, individuals being obese but fit had a lower mortality than normal weight unfit men and women [38,39]. An important issue for the future, however, is to study whether the effects on disease risk and longevity may differ between changes in absolute and relative CRF.

### 4.3. Sex- and Age-Related Annual Changes in CRF

Regression models showed a significantly larger annual decrease in CRF in men compared to women in other professionals, associate professionals, administrative and customer service and transport. Previous research has reported high variability in the sex-specific decrease of CRF in different countries, however, globally, a slightly greater decrease in CRF has been reported in women compared with men [15]. Furthermore, between 2001 and 2006, a similar increase in self-reported insufficient physical activity for both women and men have been reported in high income countries, showing similarities with our data, but not explaining the differences between men and women [40]. There was a larger decrease in younger compared with older participants, which is in accordance with earlier research [14,15], with a more pronounced difference between age-groups in white-collar occupational groups compared with blue-collar occupational groups, indicating a continuation of decreasing fitness in future older age-groups and also an increase in future adiposity, weight gain, obesity, and health-care costs [41,42,43].

### 4.4. Prevalence of Low CRF

There were both greater increases in, and higher absolute levels of, the proportion with low CRF in blue-collar compared with white-collar, and in low-skilled compared with high-skilled occupations. In low-skilled white-collar, high-skilled blue-collar, and low-skilled blue-collar, almost half had low CRF at the end of the study period. Together with the previously reported higher prevalence of daily smoking, poorer diet, no regular exercise, and a higher risk of myocardial infarction in these groups compared with high-skilled white-collar occupations [10,44], this highlights the need for targeted lifestyle interventions in these occupational groups for future health and sustainability. Solely in relation to CRF, the annual difference in total and cardiovascular disease related health care costs has been reported to be double (41% and 56% higher) in the group with low CRF (mean 29.4 mL/min/kg) and high CRF (mean 45.5 mL/min/kg) [43]. In the present study, blue-collar low-skilled workers had the lowest mean CRF and the highest prevalence of low CRF—an occupational group which has previously been shown to have the most strenuous physical working situation but the lowest level of leisure time exercise [10]. This combination of low CRF and high physical work demands is deleterious and is suggested to both impair work productivity [11,45], as well as to increase the risk of cardiovascular and all-cause mortality compared with individuals with low physical work demands [46]. On the other hand, in individuals with low CRF, even a small increase in exercise level can lead to an increase in CRF, compared with well-trained individuals, where a large amount of extra exercise is usually required for a small change of CRF [1].

### 4.5. Future Projections

We report a possible future decrease in CRF in all aggregated occupational groups, with a greater projected decrease in low-skilled compared with high-skilled occupational groups. This might have importance for future morbidity and mortality risk. For example, the risk for all-cause mortality and cardiovascular morbidity was 2.3% and 2.6% lower per each higher mL/min/kg of CRF in the adjusted analyses [47], which can be compared with the projected decrease of 3 mL/min/kg until 2040 in low-skilled occupational groups. Other studies have shown an even greater increase in risk, 4.3% per mL/min/kg, for all-cause mortality [48]. In addition, according to the projections, mean CRF for low-skilled occupational groups will be closer to 30 mL/min/kg, which corresponds to fitness level, which makes a brisk walk at a pace of 6 km/h or a slow jog at 7–8 km/h highly intense. This might discourage individuals to perform these easily accessible forms of exercise.

### 4.6. Future Agenda

An increase in the minimum level of physical activity recommendations (150 min of MVPA/week) would theoretically result in a reduction in working-age mortality, morbidity, and an increase in productivity, mainly through lower presenteeism and sickness absence [49]. However, occupational physical activity constitutes in general of too low intensity to have any greater impact on CRF level. However, to only add extra leisure time exercise may not be feasible in individuals who are exhausted after a full working day of movement and manual labor. Rather, for possible further health gains and lower presenteeism and sickness absence in relation to both occupational and leisure time physical activity, designing work tasks according to the Goldilocks principle have been suggested [50]. The idea of the Goldilocks principle is to get the right amount of physical activity, sedentary behavior, and recovery at work for each worker to improve their health and physical capacity [51]. This could be achieved through structural changes at work. It could be changes in the variety of work tasks performed or through exercise at work. For instance, a training intervention where cleaners with a high physical work load and low CRF exercised one day a week at work, increased their CRF and therein their physical work capacity, making the work relatively less straining [52]. Occupational groups with physically demanding work situations seem to be those that have a larger negative health impact of low CRF compared with those in less physically demanding occupations [46]. In the present study, blue-collar low-skilled occupational groups had the highest prevalence of low CRF, and were at the same time the group with, in general, the most physically demanding work. This makes them a high priority target for future health enhancing interventions [10]. Therefore, it would be advantageous to promote health enhancing physical activity at work, and to diversify the physical activity pattern at work through structural changes of how tasks are performed in all occupational groups.

### 4.7. Strengths and Limitations

A limitation is that CRF was estimated with a submaximal cycle ergometer test in comparison with using direct measurement during maximal effort. However, the test has shown to be a valid and reliable estimate of CRF [18] and is commonly used in different health screenings. Another weakness is that there could be miss-categorization in occupational groups, which probably would dilute the effects of CRF by occupational group. However, the large sample size included in the study will allow for the detection of important differences and trends. The data could be subjected to selection bias as the HPA is only offered to employees connected to health care services, and the submaximal test to estimate VO_2_max is voluntary and is not offered at all workplaces with HPA. The strengths of this study include its large sample size of continuous standardized CRF assessments over the last 20 years.

## 5. Conclusions

CRF declined in most occupational groups, where administrative and customer service, mechanical manufacturing, education and transport occupations experienced the largest decrease. Men and younger individuals (18–34 years) had, in general, a more pronounced decrease in CRF. The proportion with low CRF increased, so that almost every second participant from blue-collar or low-skilled occupations had a low CRF at the end of the study period. Forecast analyses revealed a continued downward trend of CRF, especially in low-skilled occupations. Structural changes, both at the workplace and in society, are needed to halt the decline in cardiorespiratory fitness.

## Figures and Tables

**Table 1:** Characteristics of the unadjusted sample divided into five-year periods.

	2001–2005	2006–2010	2011–2015	2016–2020
*n*	71,309	117,600	175,963	151,250
Women	52%	47%	40%	35%
Men	48%	53%	60%	65%
18–34 years	26%	24%	25%	29%
35–49 years	43%	44%	45%	40%
50–65 years	31%	33%	31%	31%
Education ≥16 years	23%	25%	28%	29%
Education 10-15 years	66%	65%	64%	65%
Education ≤9 years	11%	10%	8%	7%
White-collar high-skilled	55%	53%	56%	52%
White-collar low-skilled	24%	21%	16%	17%
Blue-collar high-skilled	8%	11%	15%	19%
Blue-collar low-skilled	13%	15%	13%	12%
(1) Managers	4%	6%	7%	9%
(2.1) Science and engineering	6%	7%	9%	7%
(2.2) Health care	3%	3%	4%	2%
(2.3) Education	5%	3%	2%	3%
(2.4) Other professionals	11%	10%	10%	11%
(3) Associate professionals	27%	24%	24%	21%
(4) Administrative and customer service	11%	9%	8%	9%
(5) Service, care, and shop sales	13%	12%	8%	8%
(7) Building and manufacturing	8%	11%	15%	19%
(8) Mechanical manufacturing	7%	9%	8%	7%
(8.3) Transport	2%	2%	2%	3%
(9) Elementary occupations	4%	4%	3%	3%
Height women (cm)	166.4 (6.0)	166.6 (6.0)	166.8 (6.1)	166.9 (6.2)
Height men (cm)	180.1 (6.6)	180.3 (6.6)	180.4 (6.7)	180.4 (6.7)
Weight women (kg)	68.3 (11.9)	69.2 (12.5)	69.7 (12.8)	70.2 (13.2)
Weight men (kg)	84.0 (12.6)	85.4 (13.2)	86.0 (13.5)	86.9 (14.2)

Data is presented as % or mean (SD). White-collar high-skilled includes major occupational group 1–3, white collar low-skilled includes major group 4–5, blue-collar high-skilled includes major group 7, and blue-collar low-skilled includes major groups 8–9.

## Data Availability

Data are owned by and can be requested from the Health Profile Institute.

## Appendix A

**Table A1:** Main effects of the linear regression model.

Occupation	Mean (95% CI)	*p*-Value	Adjusted *p*-Value
(1) Managers	0.000 (−0.017 to 0.018)	0.962	1.000
(2.1) Science and engineering	−0.037 (−0.056 to −0.019)	0.000	0.000
(2.2) Health care	0.046 (0.014 to 0.077)	0.005	0.180
(2.3) Education	−0.093 (−0.117 to −0.068)	0.000	0.000
(2.4) Other professionals	−0.051 (−0.065 to −0.037)	0.000	0.000
(3) Associate professionals	−0.036 (−0.046 to −0.027)	0.000	0.000
(4) Administrative and customer service	−0.105 (−0.119 to −0.091)	0.000	0.000
(5) Service, care, and shop sales	−0.059 (−0.073 to −0.045)	0.000	0.000
(7) Building and manufacturing	−0.117 (−0.130 to −0.105)	0.000	0.000
(8) Mechanical manufacturing	−0.115 (−0.131 to −0.098)	0.000	0.000
(8.3) Transport	−0.162 (−0.190 to −0.134)	0.000	0.000
(9) Elementary occupations	−0.103 (−0.127 to −0.079)	0.000	0.000

**Table A2:** Interaction effect linear regression model according to sex.

Occupation	Sex	Mean (95% CI)	*p*-Value	Adjusted *p*-Value
(1) Managers	Female	0.027 (−0.004 to 0.058)	0.090	1.000
(1) Managers	Male	−0.012 (−0.033 to 0.009)	0.282	1.000
(2.1) Science and engineering	Female	−0.036 (−0.071 to −0.001)	0.044	1.000
(2.1) Science and engineering	Male	−0.038 (−0.060 to −0.016)	0.001	0.036
(2.2) Health care	Female	0.065 (0.028 to 0.103)	0.001	0.036
(2.2) Health care	Male	−0.003 (−0.061 to 0.055)	0.928	1.000
(2.3) Education	Female	−0.089 (−0.118 to −0.061)	0.000	0.000
(2.3) Education	Male	−0.102 (−0.149 to −0.056)	0.000	0.000
(2.4) Other professionals	Female	−0.018 (−0.036 to 0.001)	0.069	1.000
(2.4) Other professionals	Male	−0.089 (−0.109 to −0.069)	0.000	0.000
(3) Associate professionals	Female	0.004 (−0.010 to 0.017)	0.605	1.000
(3) Associate professionals	Male	−0.070 (−0.083 to −0.058)	0.000	0.000
(4) Administrative and customer service	Female	−0.065 (−0.082 to −0.047)	0.000	0.000
(4) Administrative and customer service	Male	−0.170 (−0.192 to −0.147)	0.000	0.000
(5) Service, care, and shop sales	Female	−0.054 (−0.070 to −0.037)	0.000	0.000
(5) Service, care, and shop sales	Male	−0.074 (−0.102 to −0.046)	0.000	0.000
(7) Building and manufacturing	Female	−0.057 (−0.110 to −0.005)	0.033	1.000
(7) Building and manufacturing	Male	−0.120 (−0.133 to −0.108)	0.000	0.000
(8) Mechanical manufacturing	Female	−0.080 (−0.117 to −0.042)	0.000	0.000
(8) Mechanical manufacturing	Male	−0.123 (−0.141 to −0.105)	0.000	0.000
(8.3) Transport	Female	0.029 (−0.073 to 0.131)	0.580	1.000
(8.3) Transport	Male	−0.176 (−0.205 to −0.148)	0.000	0.000
(9) Elementary occupations	Female	−0.093 (−0.123 to −0.062)	0.000	0.000
(9) Elementary occupations	Male	−0.118 (−0.154 to −0.081)	0.000	0.000

**Table A3:** Interaction effect linear regression model according to age-group.

Occupation	Age-Group (yrs)	Mean (95% CI)	*p*-Value	Adjusted *p*-Value
(1) Managers	18–34	−0.076 (−0.132 to −0.020)	0.008	0.288
(1) Managers	35–49	−0.051 (−0.075 to −0.026)	0.000	0.000
(1) Managers	50–65	0.085 (0.057 to 0.113)	0.000	0.000
(2.1) Science and engineering	18–34	−0.081 (−0.114 to −0.048)	0.000	0.000
(2.1) Science and engineering	35–49	−0.049 (−0.077 to −0.022)	0.001	0.036
(2.1) Science and engineering	50–65	0.047 (0.009 to 0.086)	0.017	0.612
(2.2) Health care	18–34	−0.058 (−0.124 to 0.008)	0.086	1.000
(2.2) Health care	35–49	0.041 (−0.007 to 0.089)	0.094	1.000
(2.2) Health care	50–65	0.117 (0.064 to 0.170)	0.000	0.000
(2.3) Education	18–34	−0.262 (−0.318 to −0.207)	0.000	0.000
(2.3) Education	35–49	−0.094 (−0.132 to −0.056)	0.000	0.000
(2.3) Education	50–65	−0.011 (−0.050 to 0.028)	0.577	1.000
(2.4) Other professionals	18–34	−0.175 (−0.203 to −0.148)	0.000	0.000
(2.4) Other professionals	35–49	−0.057 (−0.078 to −0.036)	0.000	0.000
(2.4) Other professionals	50–65	0.059 (0.034 to 0.084)	0.000	0.000
(3) Associate professionals	18–34	−0.084 (−0.103 to −0.066)	0.000	0.000
(3) Associate professionals	35–49	−0.065 (−0.079 to −0.051)	0.000	0.000
(3) Associate professionals	50–65	0.043 (0.027 to 0.060)	0.000	0.000
(4) Administrative and customer service	18–34	−0.199 (−0.223 to −0.175)	0.000	0.000
(4) Administrative and customer service	35–49	−0.110 (−0.132 to −0.087)	0.000	0.000
(4) Administrative and customer service	50–65	0.009 (−0.017 to 0.035)	0.493	1.000
(5) Service, care, and shop sales	18–34	−0.094 (−0.120 to −0.068)	0.000	0.000
(5) Service, care, and shop sales	35–49	−0.070 (−0.093 to −0.048)	0.000	0.000
(5) Service, care, and shop sales	50–65	−0.018 (−0.041 to 0.006)	0.144	1.000
(7) Building and manufacturing	18–34	−0.136 (−0.157 to −0.114)	0.000	0.000
(7) Building and manufacturing	35–49	−0.135 (−0.155 to −0.115)	0.000	0.000
(7) Building and manufacturing	50–65	−0.072 (−0.095 to −0.048)	0.000	0.000
(8) Mechanical manufacturing	18–34	−0.153 (−0.181 to −0.125)	0.000	0.000
(8) Mechanical manufacturing	35–49	−0.117 (−0.143 to −0.092)	0.000	0.000
(8) Mechanical manufacturing	50–65	−0.064 (−0.095 to −0.032)	0.000	0.000
(8.3) Transport	18–34	−0.288 (−0.338 to −0.238)	0.000	0.000
(8.3) Transport	35–49	−0.110 (−0.155 to −0.066)	0.000	0.000
(8.3) Transport	50–65	−0.107 (−0.157 to −0.057)	0.000	0.000
(9) Elementary occupations	18–34	−0.165 (−0.211 to −0.120)	0.000	0.000
(9) Elementary occupations	35–49	−0.084 (−0.123 to −0.045)	0.000	0.000
(9) Elementary occupations	50–65	−0.077 (−0.116 to −0.038)	0.000	0.000

**Table A4:** Contrasts between sex.

Occupation	Sex	Mean (95% CI)	*p*-Value	Adjusted *p*-Value
(1) Managers	Female—Male	0.039 (0.001 to 0.076)	0.044	1.000
(2.1) Science and engineering	Female—Male	0.002 (−0.039 to 0.043)	0.908	1.000
(2.2) Health care	Female—Male	0.068 (−0.001 to 0.137)	0.053	1.000
(2.3) Education	Female—Male	0.013 (−0.041 to 0.068)	0.639	1.000
(2.4) Other professionals	Female—Male	0.071 (0.044 to 0.099)	0.000	0.000
(3) Associate professionals	Female—Male	0.074 (0.055 to 0.092)	0.000	0.000
(4) Administrative and customer service	Female—Male	0.105 (0.077 to 0.133)	0.000	0.000
(5) Service, care, and shop sales	Female—Male	0.020 (−0.012 to 0.052)	0.215	1.000
(7) Building and manufacturing	Female—Male	0.063 (0.009 to 0.117)	0.023	0.552
(8) Mechanical manufacturing	Female—Male	0.043 (0.002 to 0.084)	0.039	0.936
(8.3) Transport	Female—Male	0.205 (0.100 to 0.311)	0.000	0.000
(9) Elementary occupations	Female—Male	0.025 (−0.022 to 0.072)	0.295	1.000

**Table A5:** Contrasts between age-groups.

Occupation	Age-Group (yrs)	Mean (95% CI)	*p*-Value	Adjusted *p*-Value
(1) Managers	(18–34)—(35–49)	−0.025 (−0.086 to 0.036)	0.427	1.000
(1) Managers	(18–34)—(50–65)	−0.161 (−0.223 to −0.098)	0.000	0.000
(1) Managers	(35–49)—(50–65)	−0.136 (−0.173 to −0.099)	0.000	0.000
(2.1) Science and engineering	(18–34)—(35–49)	−0.032 (−0.075 to 0.011)	0.145	1.000
(2.1) Science and engineering	(18–34)—(50–65)	−0.129 (−0.179 to −0.078)	0.000	0.000
(2.1) Science and engineering	(35–49)—(50–65)	−0.097 (−0.145 to −0.049)	0.000	0.000
(2.2) Health care	(18–34)—(35–49)	−0.099 (−0.181 to −0.017)	0.017	0.612
(2.2) Health care	(18–34)—(50–65)	−0.175 (−0.260 to −0.090)	0.000	0.000
(2.2) Health care	(35–49)—(50–65)	−0.076 (−0.147 to −0.005)	0.036	1.000
(2.3) Education	(18–34)—(35–49)	−0.168 (−0.236 to −0.101)	0.000	0.000
(2.3) Education	(18–34)—(50–65)	−0.251 (−0.319 to −0.184)	0.000	0.000
(2.3) Education	(35–49)—(50–65)	−0.083 (−0.137 to −0.029)	0.003	0.108
(2.4) Other professionals	(18–34)—(35–49)	−0.118 (−0.152 to −0.084)	0.000	0.000
(2.4) Other professionals	(18–34)—(50–65)	−0.234 (−0.271 to −0.197)	0.000	0.000
(2.4) Other professionals	(35–49)—(50–65)	−0.116 (−0.148 to −0.084)	0.000	0.000
(3) Associate professionals	(18–34)—(35–49)	−0.020 (−0.043 to 0.003)	0.090	1.000
(3) Associate professionals	(18–34)—(50–65)	−0.128 (−0.152 to −0.103)	0.000	0.000
(3) Associate professionals	(35–49)—(50–65)	−0.108 (−0.129 to −0.086)	0.000	0.000
(4) Administrative and customer service	(18–34)—(35–49)	−0.090 (−0.123 to −0.057)	0.000	0.000
(4) Administrative and customer service	(18–34)—(50–65)	−0.208 (−0.243 to −0.173)	0.000	0.000
(4) Administrative and customer service	(35–49)—(50–65)	−0.118 (−0.153 to −0.084)	0.000	0.000
(5) Service, care, and shop sales	(18–34)—(35–49)	−0.024 (−0.057 to 0.010)	0.172	1.000
(5) Service, care, and shop sales	(18–34)—(50–65)	−0.076 (−0.111 to −0.041)	0.000	0.000
(5) Service, care, and shop sales	(35–49)—(50–65)	−0.053 (−0.085 to −0.021)	0.001	0.036
(7) Building and manufacturing	(18–34)—(35–49)	−0.000 (−0.029 to 0.029)	0.980	1.000
(7) Building and manufacturing	(18–34)—(50–65)	−0.064 (−0.096 to −0.032)	0.000	0.000
(7) Building and manufacturing	(35–49)—(50–65)	−0.063 (−0.095 to −0.032)	0.000	0.000
(8) Mechanical manufacturing	(18–34)—(35–49)	−0.036 (−0.073 to 0.002)	0.063	1.000
(8) Mechanical manufacturing	(18–34)—(50–65)	−0.089 (−0.131 to −0.047)	0.000	0.000
(8) Mechanical manufacturing	(35–49)—(50–65)	−0.054 (−0.094 to −0.013)	0.009	0.324
(8.3) Transport	(18–34)—(35–49)	−0.178 (−0.244 to −0.111)	0.000	0.000
(8.3) Transport	(18–34)—(50–65)	−0.181 (−0.252 to −0.110)	0.000	0.000
(8.3) Transport	(35–49)—(50–65)	−0.003 (−0.070 to 0.063)	0.921	1.000
(9) Elementary occupations	(18–34)—(35–49)	−0.082 (−0.141 to −0.023)	0.007	0.252
(9) Elementary occupations	(18–34)—(50–65)	−0.089 (−0.148 to −0.029)	0.003	0.108
(9) Elementary occupations	(35–49)—(50–65)	−0.007 (−0.061 to 0.048)	0.803	1.000
